# Genotyping-by-sequencing of three mapping populations for identification of candidate genomic regions for resistance to sterility mosaic disease in pigeonpea

**DOI:** 10.1038/s41598-017-01535-4

**Published:** 2017-05-12

**Authors:** Rachit K. Saxena, Sandip M. Kale, Vinay Kumar, Swathi Parupali, Shourabh Joshi, Vikas Singh, Vanika Garg, Roma R. Das, Mamta Sharma, K. N. Yamini, Anuradha Ghanta, Abhishek Rathore, C. V. Sameerkumar, K. B. Saxena, Rajeev K. Varshney

**Affiliations:** 1 International Crops Research Institute for the Semi-Arid Tropics (ICRISAT), Patancheru, 502 324 India; 2 Institute of Biotechnology, Professor Jayshankar Telangana State Agricultural University (PJTSAU), Rajendranagar, Hyderabad 500 030 India; 3 School of Plant Biology and Institute of Agriculture, The University of Western Australia, Crawley, WA 6009 Australia

## Abstract

Sterility mosaic disease (SMD) is one of the serious production constraints that may lead to complete yield loss in pigeonpea. Three mapping populations including two recombinant inbred lines and one F_2_, were used for phenotyping for SMD resistance at two locations in three different years. Genotyping-by-sequencing approach was used for simultaneous identification and genotyping of SNPs on above mentioned populations. In total, 212,464, 89,699 and 64,798 SNPs were identified in ICPL 20096 × ICPL 332 (PRIL_B), ICPL 20097 × ICP 8863 (PRIL_C) and ICP 8863 × ICPL 87119 (F_2_) respectively. By using high-quality SNPs, genetic maps were developed for PRIL_B (1,101 SNPs; 921.21 cM), PRIL_C (484 SNPs; 798.25 cM) and F_2_ (996 SNPs; 1,597.30 cM) populations. The average inter marker distance on these maps varied from 0.84 cM to 1.65 cM, which was lowest in all genetic mapping studies in pigeonpea. Composite interval mapping based QTL analysis identified a total of 10 QTLs including three major QTLs across the three populations. The phenotypic variance of the identified QTLs ranged from 3.6 to 34.3%. One candidate genomic region identified on CcLG11 seems to be promising QTL for molecular breeding in developing superior lines with enhanced resistance to SMD.

## Introduction

Pigeonpea [*Cajanus cajan* (L.) Millspaugh] is the sixth most important legume crop grown predominantly in the tropical and sub-tropical regions of the world. It is a protein rich (20–23%), versatile crop and thus an important source of income for smallholder farmers. Along with that, it helps to increase soil fertility by fixing atmospheric nitrogen. India ranks first in both area under cultivation (5.06 Mha) and in production (3.29 Mt) (http://faostat3.fao.org/home/, as of August 2016), however, over the last six decades, there is not much increase in the crop yield and its productivity has remained less than one ton/ha. This is predominantly because of susceptibility of majority of cultivated varieties to various biotic stresses such as sterility mosaic disease (SMD) and *Fusarium* wilt (FW). SMD is caused by pigeonpea sterility mosaic virus (PPSMV) that is transmitted by a mite (*Aceria cajani*) and FW is caused by a fungus *Fusarium udum*. Annual losses due to SMD and FW diseases have been reported to be US$ 113 million^1^. SMD is characterized by mosaic symptoms on leaves, excessive vegetative growth and cessation of reproductive organs resulting in the sterility of the plant^2^. Since its emergence, SMD has huge negative effect on pigeonpea productivity^3^. Although, application of sprays in order to control mite populations can limit spread of the disease, identification and introgression of genomic segments attributing disease resistance through genomics-assisted breeding (GAB) programmes would be an important strategy for development of disease resistant pigeonpea varieties.

Prior to availability of the pigeonpea draft genome sequence, pigeonpea crop was considered as an orphan crop as very few genetic and genomic resources were available^4^. Moreover, low level of genetic diversity in the primary gene pool further made it challenging to develop genomic resources in pigeonpea^1, 4, 5^. As a result, limited numbers of genetic maps were available in pigeonpea^6^. Although efforts were made to identify molecular markers associated with SMD resistance^5, 7, 8^, so far only one study has reported quantitative trait loci (QTLs) for SMD resistance in pigeonpea^9^. However, the availability of the genome sequence coupled with advances in the next generation sequencing (NGS) technology has enhanced the pace of pigeonpea genomics and genetics research^10^. As a result, large-scale genomic resources have been developed in pigeonpea^11^. Also ample information on single nucleotide polymorphism (SNP) has been generated in pigeonpea lines from the cultivated gene pool. SNPs are more advantageous than many other markers due to their high abundance in the genome, ease of automation in genotyping and ubiquitous distribution throughout the genome. Very recently, sequencing-based trait mapping approach has been utilized for the identification of SNPs associated with resistance to SMD and FW resistance through re-sequencing of extreme pools of selected RILs along with the resistant parent^12^. In addition, a number of new approaches such as restriction site associated DNA sequencing (RADseq)^13^, genotyping-by-sequencing (GBS)^14^, etc. have also been developed for simultaneous SNP discovery and genotyping of thousands of samples in cost effective manner in several crops. Among different approaches, GBS has been considered as the simplest and cost effective approach because of its simple library preparation procedure and high level multiplexing capacity^14^. GBS approach has been used widely for diversity studies, trait mapping and genome-wide association studies (GWAS)^15^ in a number of crops like chickpea^16^, common bean^17^, wheat^18^, cabbage^19^, etc.

In the present study, GBS approach was used for SNP identification and genotyping of three different mapping populations segregating for SMD. The identified SNPs were used in construction of high density genetic maps for three populations. The SNP genotyping data together with multi-location and multi-year phenotyping data on these populations were used to identify QTLs governing SMD resistance in pigeonpea.

## Results

### Phenotypic evaluation

**Figure 1:**
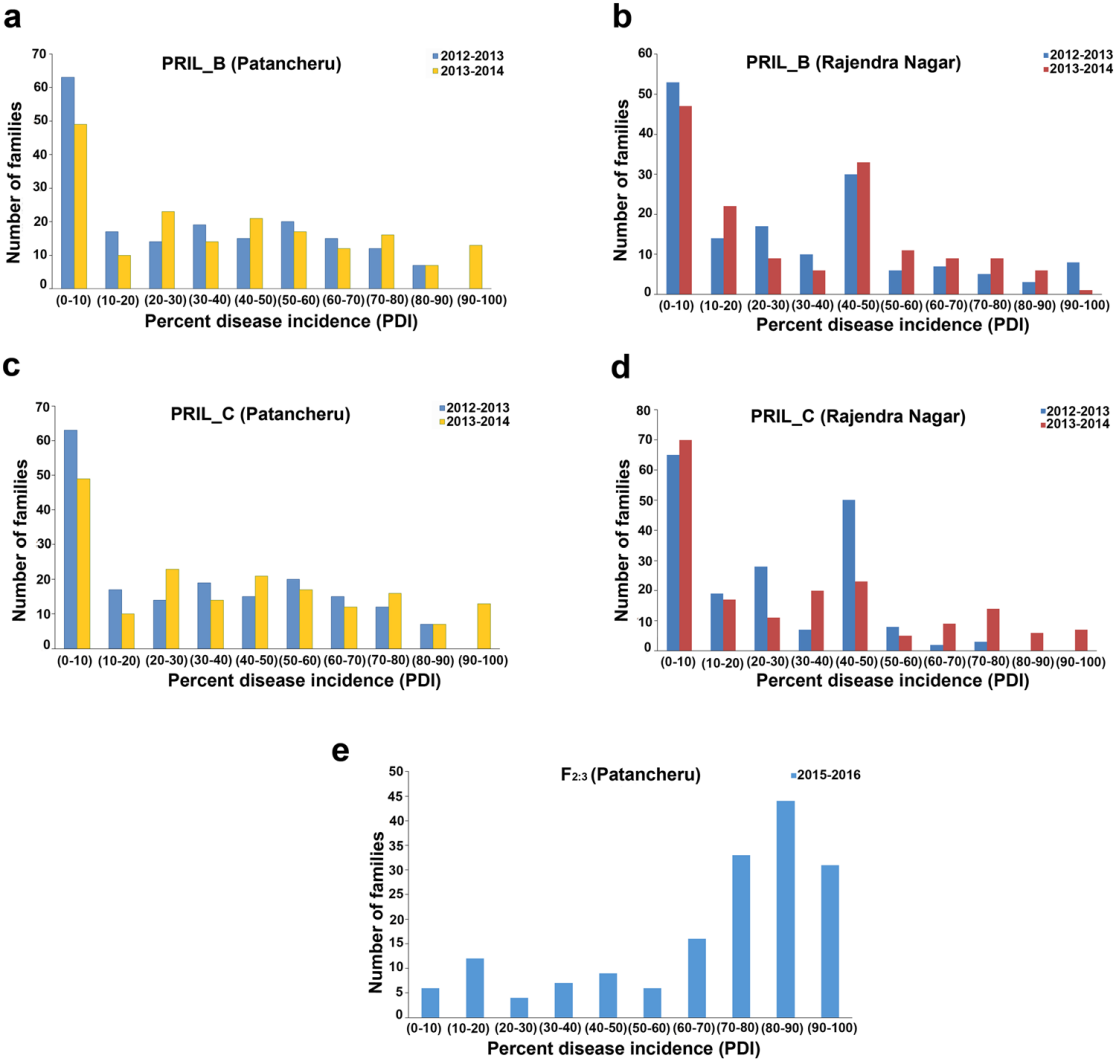
Frequency distribution of percent disease incidence (PDI) for Patancheru SMD isolate in various populations at different locations and years. The disease scoring was done on the basis of percentage of affected plants wherein 0% means complete resistance while 100% means complete susceptibility to SMD. The PDI was monitored for two consecutive years (2012–2013, 2013–2014) in ICPL 20096 × ICPL 332 (PRIL_B) and ICPL 20097 × ICP 8863 (PRIL_C) population while for one year (2015–2016) in ICP 8863 × ICPL 87119 (F_2_) population. The PDI was divided into 10 categories and number of families falling in each category were plotted as bar plot. The PDI in ICPL 20096 × ICPL 332 (PRIL_B) population at Patancheru location and Rajendranagar, Hyderabad location is shown in **a** and **b**. Figure **c** and **d** represent PDI in ICPL 20097 × ICP 8863 (PRIL_C) population at Patancheru and Rajendranagar, Hyderabad location respectively, while, the Figure **e** represents the PDI in ICP 8863 × ICPL 87119 (F_2_) population at Patancheru location.

The cumulative disease occurrence at 90 days of sowing was considered as the final percent diseases incidence (PDI). The PDI on the PRIL_B population ranged from 0 to 100 at both Patancheru and Rajendranagar, Hyderabad location (Supplementary Table 1). The parents of the mapping population showed contrasting phenotypic reaction for SMD resistance. ICPL 20096 exhibited mean PDI score of 2.08 and 5.00 at Patancheru and Rajendranagar, Hyderabad locations respectively, whereas susceptible parent ICPL 332 showed PDI score of 100 at both Patancheru and Rajendranagar, Hyderabad locations. Similarly, in PRIL_C, the PDI score ranged from 0 to 100 at both Patanchru and Rajendranagar, Hyderabad locations (Supplementary Table 1) wherein the mean PDI score of resistant parent ICPL 20097 was 0 and of susceptible parent ICP 8863 was 100 and 66.67 at Patancheru and Rajendranagar, Hyderabad locations respectively. In the case of F_2:3_ population, the mean PDI score ranged from 5.1 to 98.2, whereas the parents exhibited 100 (ICP 8863) and 0 (ICPL 87119) PDI score at Patancheru location. The frequency distributions corresponding to each of the populations are presented in the histograms (Fig. 1), which indicate the presence of several genes governing SMD resistance. Higher numbers of lines were found to be resistant in case of both the PRILs whereas, susceptibility was found to be predominant in F_2:3_ population, indicating the role of different genetic factors contributing to SMD resistance in pigeonpea. The arcsine transformed means of SMD were considered for QTL analysis in order to avoid distortion abnormalities.

### SNP discovery and genotyping

**Figure 2:**
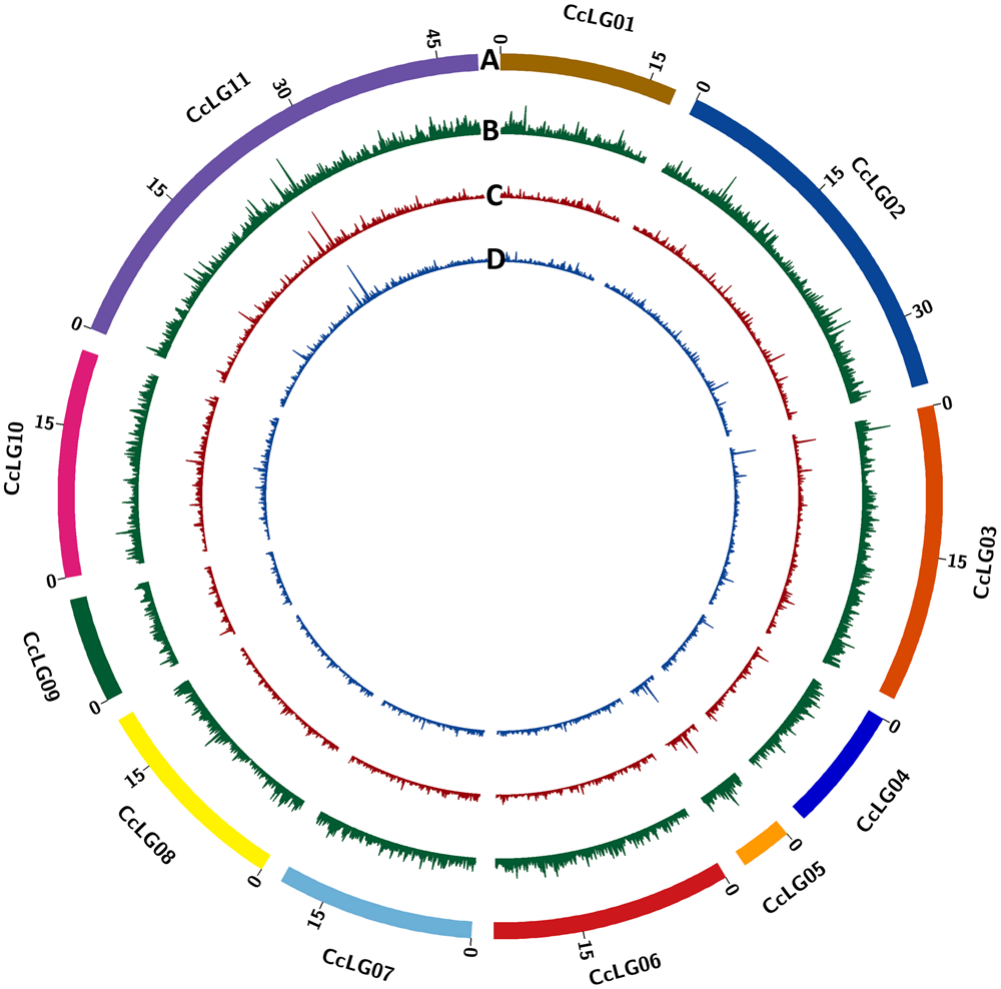
Genome-wide distribution of SNPs identified in ICPL 20096 × ICPL 332 (PRIL_B), ICPL 20097 × ICP 8863 (PRIL_C) and ICP 8863 × ICPL 87119 (F_2_) populations in pigeonpea. The number of SNPs identified within 100 Kb interval were calculated and plotted as a smooth line curve. The height of the curve is proportional to the number of SNPs within that 100 Kb interval. (**A**) Pigeonpea pseudomolecules, labelled as CcLG01 to CcLG11 and each pseudomolecule is shown in different colour. The numbers on arches represent the scale for the size of pseudomolecule in Mb. (**B**) Genome-wide distribution of SNPs identified in ICPL 20096 × ICPL 332 (PRIL_B) population (**C**) Genome-wide distribution of SNPs identified in ICPL 20097 × ICP 8863 (PRIL_C) population and (**D**) Genome-wide distribution of SNPs identified in ICP 8863 × ICPL 87119 (F_2_) population in pigeonpea.

High throughput sequencing of three mapping populations and parental lines on HiSeq 2500 platform provided large amount of sequencing data. For instance, 34.37 Gb (340.30 million reads; Supplementary Table 2), 42.32 Gb (419.04 million reads; Supplementary Table 3) and 28.76 Gb (284.77 million reads; Supplementary Table 4) data were generated for PRIL_B, PRIL_C and F_2_ respectively. For all three populations, samples having less than 80 Mb data were discarded from further analysis in order to reduce missing data error. The number of reads obtained within a given population varied from 0.80 million to 5.46 million, 0.80 million to 5.43 million and 0.84 million to 8.19 million in PRIL_B, PRIL_C and F_2_ respectively (Supplementary Fig. 1). Further, filtered sequencing reads in each population were analysed separately for SNP identification using TASSEL-GBS pipeline. As a result 212,464; 89,699 and 64,798 SNPs were identified in PRIL_B, PRIL_C and F_2_ populations, respectively. The SNP density on each pseudomolecule or chromosome has been represented using circos diagram (Fig. 2).

Considering the different levels of heterozygosity in PRILs and F_2_ populations, different criteria were used to filter SNPs identified in these populations. For PRIL_B and PRIL_C, the SNPs with ≥50% missing data and minor allele frequency (MAF) of ≤0.3 were filtered out. Additionally, lines in PRILs having more than 50% missing data were removed. Further, imputation of missing data was carried out using FSFHap algorithm^20^ implemented in TASSEL v4.0. This resulted in the identification of 1,789 SNPs in 153 lines of PRIL_B and 507 SNPs in 182 lines of PRIL_C (Table 1). The number of SNPs identified ranged from 26 (CcLG05) to 427 (CcLG11) in PRIL_B while those in PRIL_C ranged from 2 (CcLG05) to 100 (CcLG02) (Table 1). In the case of the F_2_ population, 3,941 SNPs with contrasting alleles in parental genotypes and having <30% missing data were retained for further study (Table 1). Identified SNPs were also scattered in all CcLGs with maximum 908 SNPs on CcLG11 and minimum 87 SNPs on CcLG05 in F_2_ population.

**Table 1:** Features of genetic maps in ICPL 20096 × ICPL 332 (PRIL_B), ICPL 20097 × ICP 8863 (PRIL_C) and ICP 8863 × ICPL 87119 (F_2_) populations in pigeonpea.

Chromosome	ICPL 20096 × ICPL 332 (PRIL_B)				ICPL 20097 × ICP 8863 (PRIL_C)				ICP 8863 × ICPL 87119 (F_2_)			
	SNPs	SNPs mapped	Distance (cM)	Avg. inter marker distance (cM)	SNPs	SNPs mapped	Distance (cM)	Avg. inter marker distance (cM)	SNPs	SNPs mapped	Distance (cM)	Avg. inter marker distance (cM)
**CcLG01**	75	24	74.07	3.09	27	21	50.88	2.42	117	40	120.00	3.00
**CcLG02**	219	139	114.43	0.82	100	99	148.51	1.50	641	178	178.10	1.00
**CcLG03**	172	123	97.53	0.79	48	48	111.63	2.33	386	81	167.00	2.06
**CcLG04**	87	52	77.68	1.49	41	27	53.70	1.99	251	19	114.40	6.02
**CcLG05**	26	10	30.26	3.03	2	2	16.00	8.00	87	18	88.00	4.89
**CcLG06**	205	119	111.05	0.93	51	51	53.21	1.04	336	75	187.00	2.49
**CcLG07**	148	69	97.64	1.42	69	69	61.18	0.89	323	74	173.00	2.34
**CcLG08**	174	94	74.65	0.79	46	45	77.49	1.72	282	49	144.00	2.94
**CcLG09**	74	22	63.60	2.89	8	7	15.06	2.15	200	5	43.90	8.78
**CcLG10**	182	67	61.91	0.92	52	52	101.23	1.95	410	136	179.90	1.32
**CcLG11**	427	382	118.39	0.31	63	63	109.36	1.74	908	321	202.00	0.63
**Total**	**1789**	**1101**	**921.21**	**0.84**	**507**	**484**	**798.25**	**1.65**	**3,941**	**996**	**1597.30**	**1.60**

### Genetic maps for three populations

**Figure 3:**
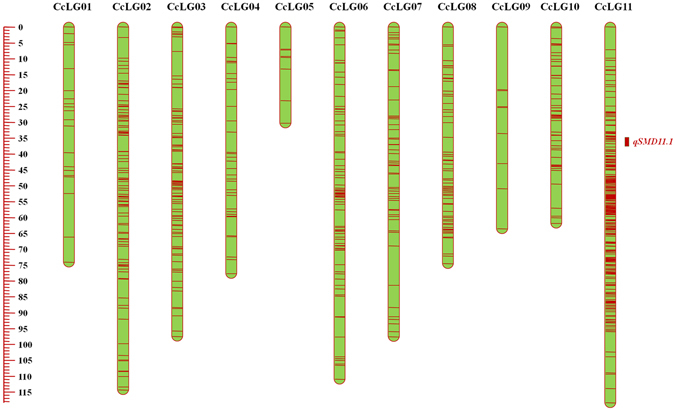
Genetic and QTL map comprising 1,101 SNPs and spanning 921.21 cM in ICPL 20096 × ICPL 332 (PRIL_B) population in pigeonpea. The scale on left side represents map distance in cM. The eleven linkage groups are shown as vertical bars and each horizontal line on the bar represent single SNP marker. Aggregation on horizontal lines indicate higher marker density on that particular linkage group. The single, consistent QTL (*qSMD11.1*) identified for SMD resistance on CcLG11 is shown by coloured rectangle.

**Figure 4:**
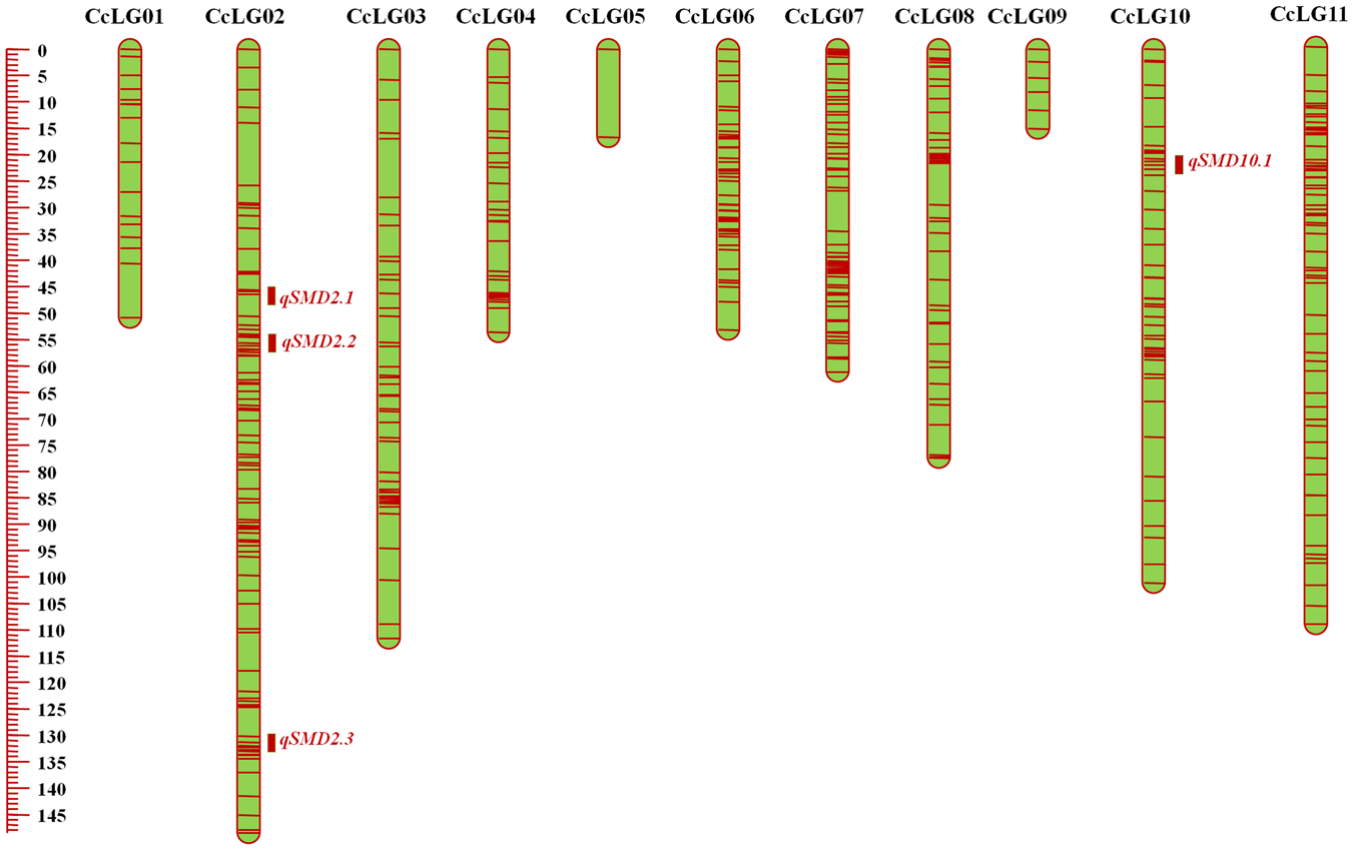
Genetic and QTL map constructed using 484 SNPs and of 798.25 cM length in ICPL 20097 × ICP 8863 (PRIL_C) population in pigeonpea. The scale on left side represents map distance in cM. The eleven linkage groups are shown as vertical bars and each horizontal line on the bar represent single SNP marker. Aggregation on horizontal lines indicate higher marker density on that particular linkage group. Three and one rectangles on right side of CcLG02 and CcLG10 represent the four QTLs identified for SMD resistance in PRIL_C population.

The imputed SNPs from PRIL_B, PRIL_C and F_2_ were used for constructing high-density genetic maps. In the case of PRIL_B population, 1,101 (61.54%) SNPs out of 1,789 SNPs were mapped on 11 linkage groups, while 484 (95.46%) SNPs out of 507 SNPs were mapped in the case of PRIL_C population (Table 1). This resulted in the construction of genetic maps of lengths 921.20 cM (Fig. 3; Table 1) and 798.25 cM (Fig. 4; Table 1) sizes in PRIL_B and PRIL_C respectively. The length of individual linkage groups varied from 30.26 cM (CcLG05) to 118.40 cM (CcLG11) in the PRIL_B population while 15.06 cM (CcLG09) to 148.51 cM (CcLG02) in the case of PRIL_C population (Table 1). A maximum of 382 (CcLG11) and 99 (CcLG02) SNPs, while minimum of 10 (CcLG05) and 2 (CcLG05) SNPs were mapped in PRIL_B and PRIL_C populations, respectively. In the case of PRIL_B population, an average inter marker distance per CcLG ranged from 0.31 cM (CcLG11) to 3.09 cM (CcLG01) with overall average of 0.84 cM (Table 1). Similarly, in PRIL_C, an average inter marker distance per CcLG ranged from 0.89 cM (CcLG07) to 8 cM (CcLG05) with overall average of 1.65 cM (Table 1).

As segregation distortion of SNPs was the major issue in F_2_ population, the strategy described by Chen *et al*.^21^ was used to filter out highly distorted markers. In this way, 996 SNPs showing expected segregation at *P* value of <10^−9^ were retained and used for construction of a genetic map of 1597.3 cM (Fig. 4; Table 1). Out of 996 SNPs, maximum 321 SNPs were mapped on CcLG11 while minimum five SNPs were mapped on CcLG09. An average inter marker distance per CcLG ranged from 0.63 cM on CcLG11 to 8.78 cM on CcLG09 with an overall average of 1.60 cM (Table 1).

### QTLs for SMD resistance

**Figure 5:**
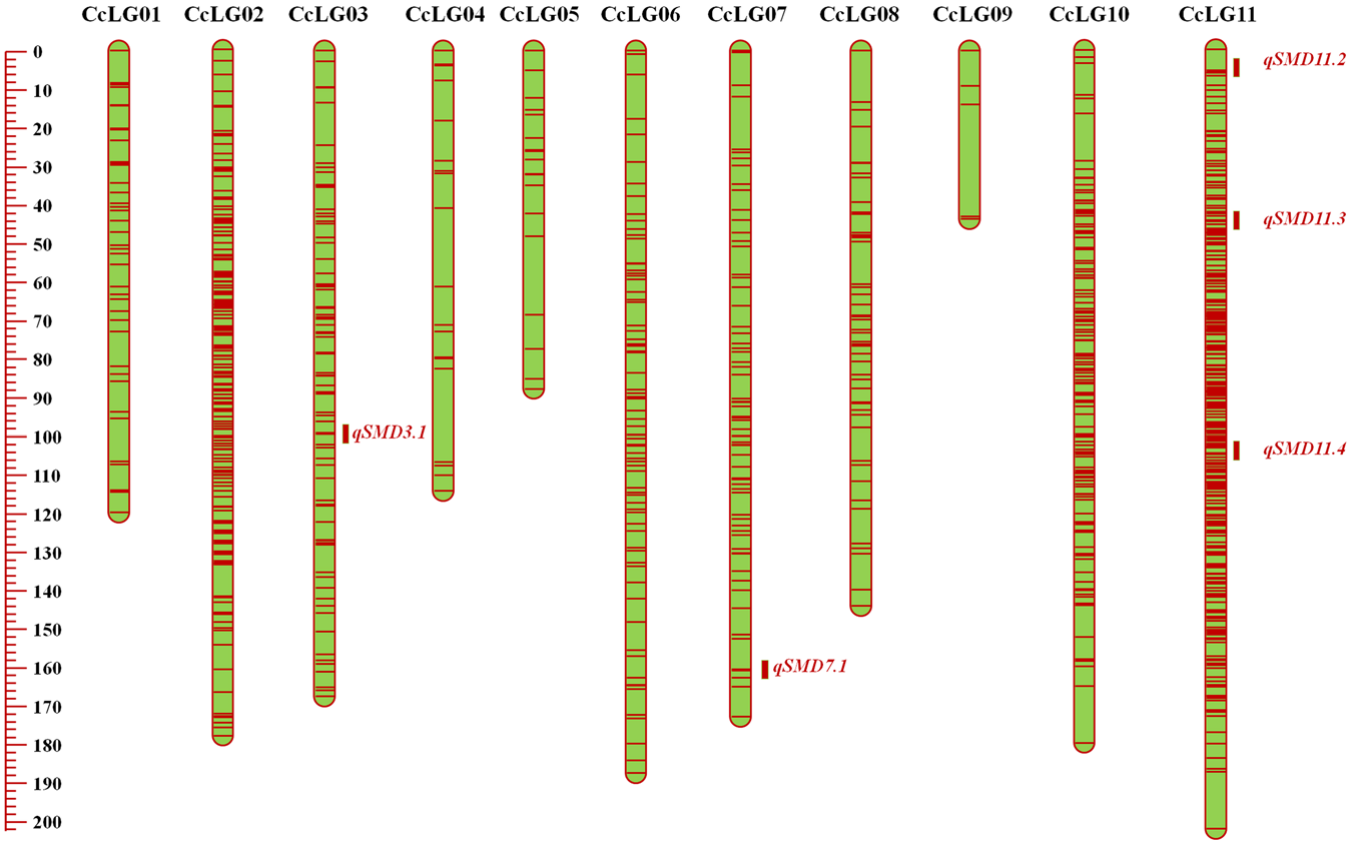
Genetic and QTL map constructed using 996 SNPs and of 1,596.30 cM length in ICP 8863 × ICPL 87119 (F_2_) population in pigeonpea. The scale on left side represents map distance in cM. The eleven linkage groups are shown as vertical bars and each horizontal line on the bar represent single SNP marker. Aggregation on horizontal lines indicate higher marker density on that particular linkage group. The QTLs identified for SMD resistance on various linkage groups have been shown as brown colored rectangle.

Phenotypic data collected for two consecutive years at two different locations for PRIL_B and PRIL_C while those collected for F_2:3_ population (for one year and one location) along with respective genotypic data were used for identification of QTLs for SMD resistance. A single consistent (appeared in more than 1 year/season) and stable (appeared in more than one location) QTL, *qSMD11.1* on CcLG11, explaining about 13% phenotypic variation (%PV) was identified in the case of PRIL_B population (Table 2; Fig. 3). In the case of PRIL_C, four QTLs were identified, out of which three were present on CcLG02 and one was present on CcLG10 (Table 3; Fig. 4). The QTL on CcLG10 (*qSMD10.1*) was found stable as well as consistent. The %PV explained by each QTL varied from 6.64 to 7.61 in which the maximum %PV was explained by the QTL from CcLG10 (*qSMD10.1*). In the case of F_2_ population, a total of five QTLs one each on CcLG03 (*qSMD3.1*) and CcLG07 (*qSMD7.1*) while three on CcLG11 (*qSMD11.2*, *qSMD11.3*, *qSMD11.4*) were identified (Table 4; Fig. 5). The %PV explained by the QTLs in F_2_ population varied from 3.6 to 34.3, wherein the QTL on CcLG03 (*qSMD3.1*) showed maximum 34.3% PV. However, no common QTL was identified among three populations for SMD resistance. Physical positions of SNPs in genome flanking the QTL region were used to determine the size of each QTL in Mb. QTLs sizes range from 1.27 to 16.66 Mb wherein the smallest QTL (*qSMD10.1*) was identified on CcLG10 in PRIL_C population while largest QTL (*qSMD2.1*) was identified on CcLG02 in PRIL_C population (Supplementary Table 5). Moreover, physical positions were also used to determine the number of genes present within each QTL region and minimum 125 genes were identified within QTL, *qSMD10.1*, on CcLG10 while maximum 1,556 genes were identified within QTL, *qSMD2.1*, on CcLG02 in PRIL_C population (Supplementary Table 5). Refinement of these QTLs regions with high density genotyping would help to identify candidate genes for SMD resistance in pigeonpea. In parallel, the positions of QTLs identified in earlier study^9^ were also determined and compared with the QTLs identified in present study to identify common QTLs. For this, the BAC-end sequences (BES)^22^ of BES-SSR markers were extracted from NCBI database and searched against the pigeonpea reference genome using blastn program. The location of the best hit obtained for each sequence was considered as location of respective BES-SSR marker. Two QTLs identified on LG 9 in ICP 8863 × ICPL 20097 were found to be located on CcLG03 pseudomolecule (Supplementary Table 6) while, the QTLs identified in TTB 7 × ICP 7035 population were found to be distributed on unassembled scaffolds of pigeonpea reference genome (Supplementary Table 6). However, no common QTL has been identified between earlier and present study.

**Table 2:** Results of QTL analysis in ICPL 20096 × ICPL 332 (PRIL_B) population in pigeonpea.

QTL	Location	Year	Chromosome	Position (cM)	Marker interval	QTL size (cM)	% PV^*^ explained	Additive effect	LOD
*qSMD11.1*	Patancheru	2012–﻿2013	CcLG11	36.81	S11_30004779 –S11_36027138	1.71	9.89	−14.46	3.07
	Patancheru	2013–2014	CcLG11	36.81	S11_30004779 –S11_36027138	1.71	9.46	−13.80	3.36
	Rajendranagar, Hyderabad	2012–2013	CcLG11	36.81	S11_30004779 –S11_36027138	1.71	12.99	−12.98	4.62
	Rajendranagar, Hyderabad	2013–2014	CcLG11	36.81	S11_30004779 –S11_36027138	1.71	9.54	−10.06	3.39

^*^%Phenotypic variation.

**Table 3:** Summary of QTL analysis in ICPL 20097 × ICP 8863 (PRIL_C) population in pigeonpea.

QTL	Location	Year	Chromosome	Position (cM)	Marker interval	QTL size (cM)	% PV^*^ explained	Additive effect	LOD
*qSMD2.1*	Patancheru	2012–2013	CcLG02	45.91	S2_16997696–S2_336378	4.4	6.64	−0.04	2.68
*qSMD2.2*	Patancheru	2012–﻿2013	CcLG02	55.61	S2_959921–S2_17370903	2.9	7.36	−0.05	2.62
*qSMD2.3*	Patancheru	2012–﻿2013	CcLG02	130.31	S2_33097589–S2_36345936	10.9	6.74	−0.03	2.81
*qSMD10.1*	Rajendranagar, Hyderabad	2013–﻿2014	CcLG10	22.81	S10_13987842–S10_15260172	5.061	7.41	0.10	3.34
	Rajendranagar, Hyderabad	2013–2014	CcLG10	22.81	S10_13987842–S10_15260172	5.061	7.61	8.10	3.47
	Patancheru	2013–2014	CcLG10	23.91	S10_13987842–S10_15260172	5.061	6.80	8.24	2.57

*%Phenotypic variation.

**Table 4:** Summary of QTLs for SMD resistance, position of the QTL on the map, percentage of phenotypic variance explained (% PV), additive and dominant effects, and LOD scores in ICP 8863 × ICPL 87119 (F2) population in pigeonpea.

QTL	Location	Year	Chromosome	Position (cM)	Marker interval	QTL size (cM)	% PV^*^ explained	Additive effect	Dominant	LOD
*qSMD3.1*	Patancheru	2015–2016	CcLG03	98.41	S3_18837756–S3_5324938	1.4	34.3	−18.4	25.7	2.8
*qSMD7.1*	Patancheru	2015–2016	CcLG07	162.21	S7_14725598–S7_7547477	9.7	14	17.7	−1.5	2.9
*qSMD11.2*	Patancheru	2015–2016	CcLG11	6.11	S11_22689650–S11_24071417	1.8	3.6	17.2	10.8	2.9
*qSMD11.3*	Patancheru	2015–2016	CcLG11	46.61	S11_16365686–S11_5757417	0.9	24.2	−14.8	16.8	5.8
*qSMD11.4*	Patancheru	2015–2016	CcLG11	104.71	S11_11799692–S11_5757598	0.4	5.2	1.8	−24.2	3

*% phenotypic variation.

## Discussion

Sterility mosaic disease (SMD) also known as “green plague of pigeonpea”, is one of the severe threats in pigeonpea production causing up to 95% yield loss^3^. Development of SMD resistant pigeonpea varieties is an important strategy for sustainable agriculture development. Marker assisted selection (MAS) has proved its importance in development of improved cultivars^23–25^, however, availability of sufficient genetic and genomic resources is the basic pre-requisite. Although, some efforts were taken in recent past to develop genetic and genomic resources in pigeonpea, limited or no success has been achieved to develop disease resistant pigeonpea cultivars through MAS. Till date, one QTL study^9^ employing SSR based linkage map and one NGS based Seq-BSA^12^ study has been carried out to identify QTLs/candidate genes for SMD resistance in pigeonpea. In this regard, genetic maps with reasonable marker-density and genomic regions associated with SMD resistance identified in the present study are important milestones in pigeonpea breeding.

### GBS for faster mapping in pigeonpea

Earlier markers based studies reported exceptionally low level of polymorphism in cultivated pigeonpea genotypes^9, 22, 26^. Therefore, limited numbers of markers were available for genetic mapping which in turn resulted in producing very low density genetic maps in cultivated pigeonpea^6, 9^. For instance, the number of SSR markers in individual component genetic maps ranged from 59 to 140 while the consensus map constructed using six component maps had only 339 markers^6^. These studies warranted the use of high-throughput genotyping for genetic mapping studies in pigeonpea. In fact, the importance of SNPs in genetic mapping in pigeonpea was already demonstrated by Saxena *et al*.^27^ wherein a genetic map with 875 PKAMs (pigeonpea KASPar assay markers) was developed. However, GBS approach can generate thousands of SNPs in faster and cost effective manner. So far, the GBS approach has not been used in the case of pigeonpea. Therefore, this is the first report of use of GBS for simultaneous identification and genotyping of SNPs in pigeonpea. Using this approach, although several thousand SNPs were identified between parental genotypes of each of three populations, stringent criteria for considering high-quality SNPs for genetic mapping restricted construction of genetic maps with few hundred loci. However, presence of high amount of missing data is an inherent problem of GBS approach^14^ and similar results have been reported in most of the GBS based linkage mapping studies^16, 19^. Nevertheless, sequencing at higher depth and optimization of sequencing and analysis processes, may provide larger number of SNPs useful for constructing genetic maps in near future.

### Better genetic maps for pigeonpea

Three genetic maps containing 484, 996 and 1,101 SNP markers with a map distance of 798.27, 1,597.30 and 921.20 cM were constructed using PRIL_C, F_2_ and PRIL_B populations, respectively. Number of markers mapped in individual genetic maps was higher than the earlier SSR based genetic maps in pigeonpea^6, 9^. The genetic map for PRIL_C population contained less markers (484) compared to other maps constructed in the present study. This may be partly attributed to the low level of diversity present between the parental lines (ICPL 20097 and ICP 8863). Earlier study using SSR markers also reported low polymorphism between ICPL 20097 and ICP 8863 genotypes^9^. The average inter-marker distance varied from 0.84 cM (PRIL_B) to 1.65 cM (PRIL_C) and were 5 to 6 fold lesser than those reported in SSR based genetic mapping studies^6, 9^. Furthermore, the average inter marker distance observed in PRIL_B (0.84 cM) was the lowest in all the genetic mapping studies conducted in pigeonpea so far^6, 9, 27^ which indicates better saturation of available genetic maps. These better maps developed in the present study will be an important resource for not only QTL identification but also in QTL cloning and identification of candidate genes. As a very small number of common markers (7) were found between genetic maps developed for the PRIL_B and PRIL_C populations, a consensus map couldn’t be developed. This might be due to the steps followed in the GBS protocol such as restriction digestion, amplicon enrichment and sequencing of genomic DNA were completely random and therefore probability of enrichment and sequencing of similar fragment from the entire sample was very less. Combined analysis of all the GBS data and then imputation of missing data will help to reduce above issues; however, the imputation methods require extensive and accurate haplotype data and could only be possible in very few plant species such as Arabidopsis, maize, rice etc. where large scale sequencing data on different set of lines are available. Nevertheless, efforts are being taken to generate such data in pigeonpea and imputation of large amount of missing data could be possible in near future with refined genome assembly.

### Distinct sources of SMD resistance in pigeonpea

Genetic maps and multi-location phenotypic data were utilized for the identification of QTLs for SMD resistance in pigeonpea. A total of 10 QTLs, one in PRIL_B, four in PRIL_C and five in F_2_ populations, were identified. Earlier, bulk segregant analysis using AFLP^5^, RAPD and SCAR^7^ markers to identify markers linked to SMD resistance in pigeonpea. Further, microsatellite markers were also used for single marker analysis^8^ to identify markers linked to SMD resistance in pigeonpea. However, none of the study reported major QTLs (with % PV > = 10) for SMD. Further, QTL analysis was carried out using early generation (F_2_ population) of PRIL_C and identified two QTLs for resistance to Patancheru SMD isolate on linkage group 9 (LG 9)^9^. On the other hand, four QTLs on two different linkage groups were identified in PRIL_C population in present study. However, no common QTL was identified between earlier and present study. This could be because of several reasons such as population type, number of markers used, robustness of phenotyping, etc. For instance, the QTLs reported in earlier study were based on the early generation (F_2_) population, using very less number of markers (120) and using only one season phenotypic data. In contrast, four fold more markers (484) and phenotypic data collected at two different locations (Patancheru and Rajendranagar, Hyderabad) for two consecutive years have been utilized for QTL identification in genetically stable RIL population (PRIL_C). Further, whole genome re-sequencing (WGRS) based bulk sergeant analysis using PRIL_B population has identified a total of four SNPs, two on CcLG02 and one each on CcLG08 and CcLG11, associated with SMD resistance in pigeonpea^12^. Out of the four SNPs, one SNP at 19,958,148 bp position on CcLG11 was found to be present within the QTL (*qSMD11.1*) region identified on CcLG11 in present study in PRIL_B population.

Interestingly, no common QTL was identified among three different populations, indicating distinct genomic regions are associated with SMD resistance in three populations. Similar observation has been made in the earlier study^9^. This could also be because of variations in marker density in three genetic maps which may lead to less number of common markers between three genetic maps. Therefore, integration of more markers is necessary to confirm the above results. Two major QTLs (*qSMD11.1* and *qSMD11.3*) identified in the present study were present on CcLG11 suggesting importance of these regions on CcLG11 in governing SMD resistance in pigeonpea. Additionally, a whole genome re-sequencing (WGRS) based Seq-BSA approach has also identified one SNP within the QTL region identified on CcLG11 in PRIL_B population supporting the present results^12^. Detailed analysis of QTLs on CcLG11 could help to understand the genetic basis of SMD resistance in pigeonpea. Moreover, these regions can be integrated into elite lines though marker assisted breeding to improve the SMD resistance. From the previous and present studies, it is clear that SMD is very complex trait and governed by various small effect QTLs and breeding strategies involving multiple parents such as multi-parent advanced generation inter cross (MAGIC) need to be employed in order to increase accumulation of all the favorable small effect QTLs into superior genotypes.

In summary, GBS was found a very effective approach to generate large scale SNP genotyping data. Based on these data, three genetic maps with better marker density and genome coverage have been developed for PRIL_B (1,101 markers with 921.20 cM), PRIL_C (484 markers with 798.25 cM) and F_2_ (996 SNP markers with 1597.30 cM) populations. This study has generated the highest saturated genetic maps so far in pigeonpea and also identified one candidate genomic region on CcLG11 associated with SMD resistance for deployment in genomics-assisted breeding.

## Materials and Methods

### Plant material

Five parental lines viz., ICPL 20096, ICPL 332, ICPL 20097, ICP 8863 and ICPL 87119 with contrasting SMD resistance were selected for development of mapping populations. Out of these lines, ICPL 20096, ICPL 20097 and ICPL 87119 were resistant to SMD while ICPL 332 and ICP 8863 were susceptible to SMD.

Two recombinant inbred lines (RILs) populations, each of 188 lines were generated by crossing ICPL 20096 × ICPL 332 (PRIL_B) and ICPL 20097 × ICP 8863 (PRIL_C)^1^ while one early generation (F_2_) mapping population of 168 lines was generated by crossing ICP 8863 × ICPL 87119 genotypes.

### Phenotypic evaluation and statistical analysis

All the three populations along with parental lines were evaluated for SMD resistance using leaf stapling technique^28^. Disease reactions for all three populations were carried out against the Patancheru isolate. The plants were artificially inoculated by stapling the infected leaves to allow the transmission of mite vector. Resistant and susceptible controls viz. ICPL 2376 and ICP 8863 were sown at regular intervals to monitor the disease infection. Disease scoring for SMD was done periodically at 30 days interval; however final cumulative data was taken at 90 days as disease can be easily distinguished as patches of bushy, pale green plants without flowers or pods by this time. The disease severity was determined on the basis of percentage of affected plants wherein 0% means complete resistance while 100% means complete susceptibility to SMD. The PRIL_B and PRIL_C populations were evaluated for SMD at two different locations viz. International Crops Research Institute for the Semi-Arid Tropics (ICRISAT), Patancheru, India and Professor Jayashankar Telangana State Agricultural University (PJTSAU), Rajendranagar, Hyderabad, India for two consecutive years (2012–2013 and 2013–2014).

SMD screening is destructive and may lead to complete loss of seeds from susceptible plants. Therefore to avoid this issue in F_2_ population, the plants were selfed to generate F_2:3_ plants which were subsequently phenotyped for SMD resistance at ICRISAT during year 2015–2016. 10 F_2:3_ plants representing each F_2_ plant were sown in two replications following randomized complete block design (RCBD). As a result, a total of 3360 F_2:3_ plants were considered for disease scoring. BLUPs were estimated from multi-locations data and the arcsine transformed values have been utilized for performing QTL analysis.

### DNA extraction and genotyping-by-sequencing (GBS)

DNA from the parental genotypes and from individual progenies were extracted using NucleoSpin Plant II kit (Macherey-Nagel, Dren, Germany). The quality and quantity of DNA was checked on 0.8% agarose gel and then using Qubit 2.0 fluorometer (Thermo Fisher Scientific Inc., USA).

For GBS approach, 10 ng genomic DNA from each sample was restriction digested using *Ape*KI (recognition site: G/CWCG) endonuclease. The digested product was ligated with uniquely barcoded adaptors using T4 DNA ligase enzyme. Such digested ligated products from each sample were mixed in equal proportion to construct the GBS libraries, which were then amplified, purified to remove excess adapters and used for sequencing on HiSeq 2500 platform (Illumina Inc, San Diego, CA, USA).

### SNP identification and genotyping

The sequence reads from raw FASTQ files were used for SNP identification and genotyping using reference based GBS analysis pipeline implemented in TASSEL v4.0^29^. Briefly, the sequencing reads were searched for perfectly matched barcode with the expected four base remnant of the enzyme cut site. The barcode containing reads were sorted, de-multiplexed according to barcode sequence and trimmed to first 64 bases starting from enzyme cut site. Further, those reads containing ‘N’ within first 64 bases were rejected. The remaining good quality reads (called as tags) were aligned against the draft genome sequence of pigeonpea^10^ using Bowtie 2 software^30^. The alignment file was then processed through GBS analysis pipeline for SNP calling and genotyping. An allele was considered only if it was supported with a minimum tag count value of 10. The SNPs identified were further filtered to remove missing data and such filtered SNPs were used for genetic mapping and QTL analysis.

### Genetic mapping and QTL analysis

The filtered SNPs from each population were used for the construction of high density genetic maps using JoinMap V4.0^31^. The Chi-square (χ^2^) values calculated for each SNP marker using Joinmap V4.0 were used to determine segregation distortion and highly distorted and unlinked markers were excluded from further analysis. The grouping and ordering of markers was carried out using regression mapping algorithm with maximum recombination frequency of 0.4 at minimum logarithm of odds (LOD) value of 3. The marker orders after incorporation of new marker was confirmed using ripple command. Finally, Kosambi mapping function was used to convert recombination fraction into map units^32^. Mapchart 2.30^33^ was used for the visualization of the linkage groups.

The genotyping data along with phenotyping data collected for each population were used for QTL analysis using QTL Cartographer V.2.5 software^34^. The composite interval mapping (CIM) method with model 6 and other default values were used for QTL identification. The empirical LOD thresholds for each trait were determined by 1,000 permutations at the *P* ≤ 0.05 level^35^. The LOD score values were used to determine the significance of QTL intervals.

## Electronic supplementary material

Supplementary tables 1-6

Supplementary Figure 1
