# Supplementary material for: Genotyping-by-sequencing of three mapping populations for identification of candidate genomic regions for resistance to sterility mosaic disease in pigeonpea
Source: Sci Rep. 2017 May 12;7:1813. doi: 10.1038/s41598-017-01535-4 (PMC5431754; doi:10.1038/s41598-017-01535-4)
Supplement: Supplementary file 2 — Supplementary Figure 1 [file 41598_2017_1535_MOESM2_ESM.doc]

# Genotyping-by-sequencing of three mapping populations for identification of candidate genomic regions for resistance to sterility mosaic disease in pigeonpea

Rachit K Saxena^1^, Sandip M Kale^1^, Vinay Kumar^1^, Swathi Parupali^1^, Shourabh Joshi^2^, Vikas Singh^1^, Vanika Garg^1^, Roma Rani Das^1^, Mamta Sharma^1^, KN Yamini^2^, Anuradha Ghanta^2^, Abhishek Rathore^1^, CV Sameerkumar^1^, KB Saxena^1^, Rajeev K Varshney^1,3,*^

^1^International Crops Research Institute for the Semi-Arid Tropics (ICRISAT), Patancheru, 502 324, India

^2^Institute of Biotechnology, Professor Jayshankar Telangana State Agricultural University (PJTSAU), Rajendranagar, Hyderabad, 500 030, India

^3^School of Plant Biology and Institute of Agriculture, The University of Western Australia, Crawley, WA, 6009 Australia

*To whom correspondence should be addressed.

Rajeev K Varshney

International Crops Research Institute for the Semi-Arid Tropics (ICRISAT), Patancheru, 502324, India

Tel: 91-40-30713305; Fax: 91-40-30713074

Email: [r.k.varshney@cgiar.org](mailto:r.k.varshney@cgiar.org)

**
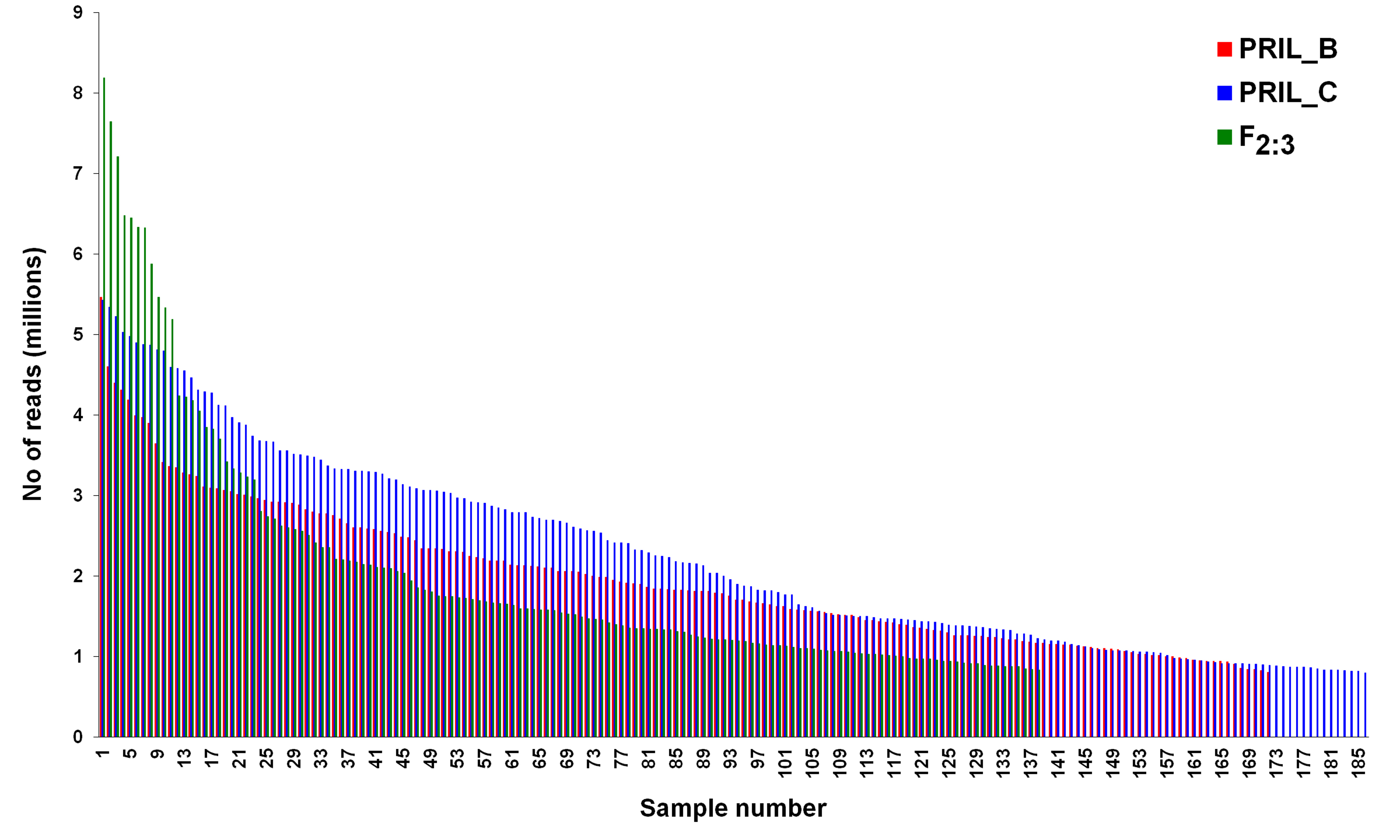
**

**Supplementary Figure 1**

Number of reads obtained for each sample in ICPL 20096 × ICPL 332 (PRIL_B), ICPL 20097 × ICP 8863 (PRIL_C) and ICP 8863 × ICPL 87119 (F_2_). The raw sequencing reads obtained were de-multiplexed according to barcode sequence and number of reads for each sample were calculated. The number of reads obtained within a given population varied from 0.80 million to 5.46 million, 0.80 million to 5.43 million and 0.84 million to 8.19 million in PRIL_B, PRIL_C and F_2_ respectively.
